# Use of 5-Enolpyruvylshikmate-3-Phosphate Synthase Encoding Gene for Typing of *Staphylococcus aureus* Isolated from Skin and Urinary Tract Infections of Human 

**Published:** 2012

**Authors:** Reza Talebi-Satlou, Malahat Ahmadi, Farokh Ghavam, Habib Dastmalchi Saei

**Affiliations:** 1 *Department of Microbiology, Faculty of Veterinary Medicine, Urmia University,Urmia, Iran*; 2 *Department of Pathology, Faculty of Medicine, University of Medical Sciences, Urmia, Iran*

## Abstract

**Objective(s):** *Staphylococcus aureus* is both a successful human commensal and a major pathogen. In this study we investigated the genetic diversity of 26 *S. aureus* isolates recovered from human skin and urinary tract infections.

**Materials and Methods:**Typing procedure for the studied *S. aureus* isolates was performed based on PCR amplification of the *aroA* gene, which encodes the enzyme 5-enolpyruvylshikmate-3-phosphate synthase (EPSPS) that involves in aromatic amino acid biosynthesis, and restriction fragment length polymorphism (RFLP) analysis of the product.

**Results:**All *S. aureus* isolates produced a single PCR amplification product of 1,153 bp. Digestion of the PCR products with the *Taq*I endonuclease revealed four different *aroA* gene patterns designated as A, B, N and H according to the nomenclature system of previous studies. In general, 80.77% of the studied isolates displayed type N, 7.69% were type B, 7.69% were type H and 3.85% displayed type A.

**Conclusion:**Divergent *aroA* types were detected among *S. aureus* isolates from skin and urinary tract infections. The results showed that urinary tract infections were contaminated by *S. aureus* isolates with identical banding patterns (A), while isolates recovered from skin infections had different *aroA* types. This study also indicates that *aroA* genotypes vary not only from region to region, but also in individual hosts within a region.

## Introduction

*Staphylococcus aureus* is an increasing public health concern because of the rapid evolution and spread of virulent/resistant clones in humans and animals. This organism is the main etiological agent and the most frequently isolated microorganism in various skin and soft tissue infections (SSTIs) reported from different parts of the world (1-6). Urinary tract infections (UTIs) are also among the most common infectious diseases diagnosed in outpatients as well as in hospitalized patients and *S. aureus* is one of the causative agents (7). The molecular mechanisms by which *S. aureus* causes skin and urinary tract infections remain poorly understood. Human skin gives physical protection partly because it is composed of tightly associated epithelial cells. These cells are covered by a highly cross-linked layer of keratin which is normally impenetrable to bacteria. Additionally, skin displays microbicidal activity via an array of bioactive molecules, among which antimicrobial peptides (AMPs) are of critical importance to host defense (8, 9). Skin fatty acids are also potent bactericidal agents and help to prevent colonization of potential pathogens. Resistance to human skin innate defenses is crucial for survival and carriage of *S. aureus*, a common cutaneous pathogen and nasal colonizer. It must be noted that specific tissue environment is decisive for the differentiation of Staphylococci (10). In a study, van Leeuwen *et al* (2005) showed that the presence (combinations) of virulence factors plays an important role in host or even tissue specificity in *S. aureus* infections (11). In a hospital setting, knowledge of the risk factors for SSTIs, clonal spread and resistance pattern are particularly useful in patient management and maintenance of infection prevention measure (12). 

There are few data concerning the genotypic characteristic of *S. aureus* obtained from skin and urinary tract infections. Population analyses based on molecular characterization have proven to be useful for establishing the relationship between clinical isolates responsible for defined diseases. On the other hand, molecular strain typing of microorganisms is now recognized as an essential component of infection control program and to trace the source of infection and routes of transmission. Many molecular methods have been used in epidemiological investigations into human staphylococcal infections such as pulsed-ﬁeld gel electrophoresis (PFGE) (13, 14), restriction fragment length polymorphism analysis (RFLP) (15, 16), random ampliﬁcation of polymorphic DNA (RAPD) (17), multilocus sequence typing (MLST) (18) and *spa* typing (19). Indeed, the sensitivity and performance of polymerase chain reaction (PCR) based typing systems make them useful tools for the study of *S. aureus* of animal and human origin, and for the monitoring of the bacterium’s diffusion. The *aroA* gene amplification has been considered a simple and rapid method for typing *S. aureus* (20). *aroA* gene encodes the enzyme 5-enolpyruvylshikmate-3-phosphate synthase (EPSPS), a key enzyme in the shikimate pathway, which catalyzes phosphoenolpyruvate (PEP) and shikimate-3-phosphate (S3P) to form 5-enolpyruvylshikmate-3-phosphate (EPSP) and inorganic phosphate. Two classes of EPSPS have been identified that share less than 50% amino acid identity (21). Class I EPSPS are naturally sensitive to glyphosate, generally identified from plants and bacteria. In contrast to class I, class II EPSPS usually has a natural tolerance of glyphosate and a high affinity for PEP. Class II EPSPS was identified from some bacteria (e.g., *Pseudomonas* sp. strain PG2982 (21), and *Agrobacterium tumefaciens* sp. strain CP4). In this study, we have utilized the *aroA* gene typing in discriminating unrelated *S. aureus* strains recovered from human urine and skin infections. 

## Materials and Methods

*Origin of the bacterial isolates*

Twenty-six *S. aureus* isolates were obtained from urinary tract (n= 16) and skin wound infections (n= 10) in Urmia region, West Azerbaijan province, Iran. The *S. aureus* strains were isolated from the site of skin infection and from the mid urine samples. The skin swabs were taken from the site of skin infection and immediately streaked on mannitol salt agar plates. After incubation for 24 hr at 37 ^○^C, colonies suspected as *S. aureus* were isolated and purified on sheep blood agar plates. The biochemical tests were used to confirm the species identification of the *S. aureus* isolates. The diagnosis of a symptomatic UTI in this study required the presence of fever >38 ^○^C and dysuria. Cytological examination of the urine required >10^4^ leucocytes/ml. Bacterial colonies from the urine were counted and the microbial flora was identified. Recovery of >10^5^ bacteria/ml from a voided specimen or >10^4^ bacteria/ml from a catheter specimen was required. Diagnosis of *S. aureus* UTI required the presence of the organism in pure or predominant (>95%) culture. 

*DNA isolation for PCR assay*

Chromosomal DNA was extracted by incubating cells grown overnight in an agar plate with Genomic DNA purification kit (Fermentas, Germany). *S. aureus* ATCC 29213 was included as a positive control in both PCR and PCR-RFLP assays. For the negative control, sterile water was added instead of nucleic acids.

*Detection of the aroA gene*

The primers used for analysis of the *aroA* gene of *S. aureus* by PCR-RFLP were constructed according to the method described by Marcos *et al* (1999) (20). The sequence of forward primer used for amplification was 5′-AAG GGC GAA ATA GAA GTG CCG GGC-3′ and the reverse primer was 5′-CAC AAG CAA CTG CAA GCA T-3′. The PCR reaction was carried out in a final volume of 50 μl reaction containing 25 μl of 2X master mix (CinnaGen, Iran), 0.8 μM of each primer, and 5 μl of template DNA. For the negative control, sterile water was added instead of nucleic acids. DNA amplification was carried out with the following thermal profile: initial denaturation at 94 °C for 2 min; 32 cycles, each consisting of a 1-min denaturation step at 92 °C, 1-min annealing step at 63 °C, and 1.5-min extension step at 72 °C (22). The reaction was completed by incubating at 72 °C for 10 min. Amplified products were separated by agarose gel electrophoresis (1.2% agarose containing 0.5 mg ethidium bromide in 0.5X TBE electrophoresis buffer) at 80 V for 1 hr and photographed under UV illuminator. 

*Molecular typing by aroA PCR-RFLP*

Enzymatic digestion of the *aroA* amplified product was performed with 5U of *Taq*I (Fermentas, Germany) as previously described (22). The resulting fragments were electrophoresed on a 1.2% agarose gel at 100 V for 1 hr, stained with ethidium bromide (0.5 µg/ml), and visualized under UV light. Based on the nomenclature scheme of previous studies (20, 22, 23) A, B, H, and N genotypes were found among the studied isolates.

## Results

**Figure 1:**
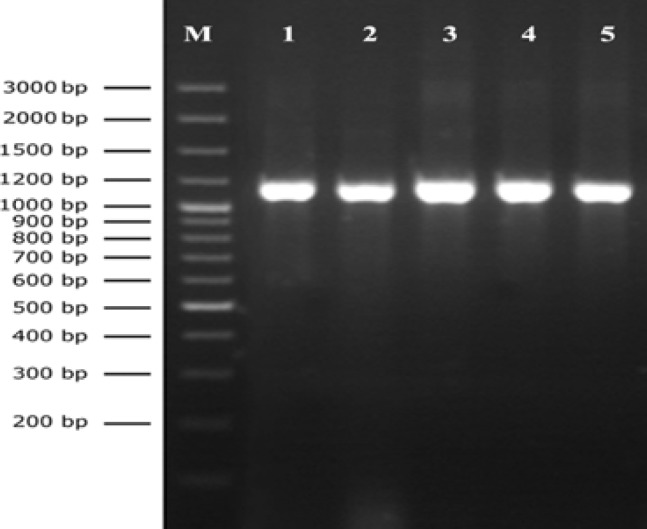
PCR-amplified *aroA* gene of selected *Staphylococcus aureus* isolates. Lane M: GeneRuler^TM^ 100 bp DNA ladder plus. Lanes 1, 2, 3, 4, and 5: amplicon of 1,153-bp

The phenotypic and biochemical properties of all 26 isolates indicated that they were all *S. aureus* strains. PCR amplification of the *aroA* gene and subsequent agarose gel electrophoresis of the amplified products showed an expected 1,153-bp amplicon for all *S. aureus* tested (Figure 1). No PCR product was amplified when sterile water was added instead of nucleic acids. 

**Figure 2:**
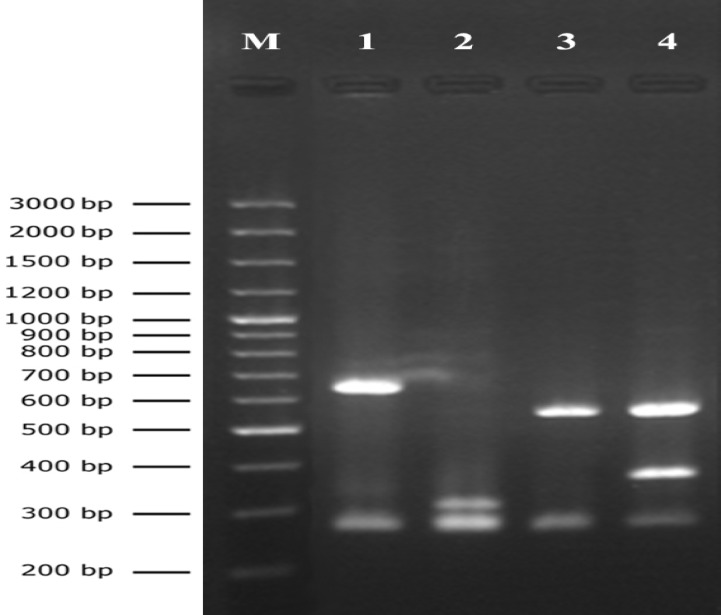
*aroA* gene restriction profiles. Lane M: GeneRuler^TM^ 100 bp DNA ladder plus. Lane 1: *aroA* pattern H. Lane 2: *aroA* pattern N. Lane 3: *aroA* pattern A. Lane 4: *aroA* pattern B

Digestion of the PCR products with the *Taq*I restriction enzyme generated four different *aroA* gene patterns (Figure 2). In total, *aroA* type N was identified in 21 out of 26 (80.77%) isolates. *aroA* types A, B, and H were observed in 1 (3.85%), 2 (7.69%), and 2 (7.69%) isolates, respectively. The *aroA* types found for the strains of *S. aureus* coming from skin and urine samples are detailed in Table 1. As shown, all of the urine originating* S. aureus* isolates showed *aroA* type N. Different *aroA* types were identified among skin isolates, from which *aroA* type N was accounted for 5 (50%) isolates.

## Discussion

Rapid identification of *S. aureus* DNA not only from bacterial cells but also from biological materials necessitates species-specific and ubiquitous nucleotide sequence as a target (24, 25). In the current study, *aroA* gene was amplified from all tested* S. aureus* isolates.

**Table 1:** The corresponding distribution of *aroA* types among isolates originating from each of the skin and urine

	No. of the isolates	*aroA* type			
		A	B	H	N
		A	B	H	N
Skin	10	1	2	2	5
Urine	16	-	-	-	16
Total	26	1	2	2	21

Marcos *et al* (1999) described a rapid, sensitive, and specific nucleic acid-based procedure that permits the identification of *S. aureus* in cows and sheep with intramammary infections by PCR amplification of the *aroA* gene (20). Since then, PCR amplification of the *aroA* gene in combination with RFLP analysis has successfully been used for *S. aureus* identification and typing (22, 23).

According to the results of the present investigation,* Taq*I digestion of the PCR-generated products from skin-related isolates revealed four different patterns A, B, H, and N, from which *aroA* type N was dominant, indicating its widespread existence. This result is in agreement with the results from Jordan, where fifty percent of the isolates collected from human specimens were designated as N (23), but is controversial when compared with earlier observation where *aroA* type N was not detected among the 59 *S. aureus* isolates from humans (20). In the present study, genotypes C and D were not detected among the tested isolates, but studies in Spain showed that 38.98% of the *S. aureus* isolates from human belonged to type A. Types B, C, and D were 13.5%, 3.3% and 44.1%, respectively (20). This is in agreement with that reported previously from the same region in Iran, where C and D *aroA* types were not detected among the bovine mastitis associated S. aureus isolates (22). These indicate that *aroA* genotypes were not distributed uniformly among various geographical areas, which may be related to different environmental conditions and the genetic background of the strain. More detailed studies including sequencing of the repeated region of the protein A gene (*spa*) to explore the area specific trait of *S. aureus* isolates will be required. As reported, the *spa* types among MRSA strains are area specific. *spa* type t002 is the most common type in Occupied Palestine (26), whereas *spa* type t041 is the common strain type in Southern Germany (27). Another well-known CA-MRSA is *spa* type t044, which is widespread in European countries (28-30). In a study from Austria, the most common *spa* types found among 382 MRSA isolates were t001, t190, t008, and t041 (31). Two *spa* types (t067 and t002) were dominant among MRSA strains in Spain (32). 

As results, skin originated *S. aureus* isolates showed more genetic diversity than the urine ones. In a study from china, MSSA strains from skin/soft tissue infections in a children’s hospital also showed diverse genetic backgrounds (33). Gadepalli *et al* (2009) also reported that different clones were responsible for all cases of SSTIs (12). 

Considering the results from the current study and *aroA* RFLP patterns reported previously by Saei *et al* (2010) among the bovine mastitis-related *S. aureus* isolates, shows that the frequency of genotypes found for isolates of *S. aureus* from cow were in contrast to those found for isolates from human. One possible explanation for this is a host- and/or tissue-specific pathogenic trait of *S. aureus* genotypes may be due to genetic background. Apparently, the nature of the virulence genes encountered in an *S. aureus* strain is primarily an important determinant for host or even tissue specificity (11). Population genetic analyses have also provided strong evidence of host specialization among *S. aureus* clonal groups associated with human and ruminant infection (34). Although studies of the molecular epidemiology of *S. aureus* strongly suggest that a genetic subset of strains is particularly well adopted for causing infection in cattle (35-37), the distinct bovine clone groups are often interspread among the human clone groups in a manner suggesting that human-associated *S. aureus* clones were the evolutionary precursors to modern bovine specialist *S. aureus* clones (38, 39).

As a result, *aroA* types A, B and H were only detected among skin associated isolates. Existence of such a divergence shows that there is a possibility of skin contamination from various sources likely due to frequent person-to-person contact, contamination of surfaces, and sharing of personal hygiene items. This may also be as a consequence of the presence of certain virulence determinants and subsequence interaction between *S. aureus* and skin defense mechanisms. The first step in the establishment of *S. aureus* infection is adhesion to host tissues. *S. aureus* nasal and skin isolates produce Fibronectin Binding Protein-A (FnbPA) at a high frequency (40). It has been shown that IsdA (iron-responsive surface determinant A) allows *S. aureus* to colonize skin by blocking the action of several antibacterial molecules present in normal skin (41). Expanded studies are necessary to trace the origin of various genotypes of *S. aureus* that infect human skin. The significance of nasal carriage of *S. aureus* as risk factor for human skin infection has been reported previously (42, 43). Tulloch (1954) reported that Staphylococci isolated from skin lesions in patients with chronic Staphylococcal skin infections were of the same phage-type as the Staphylococci isolated from anterior nares of the patients (44). MRSA nasal carriage, identified in 0.2% to 2.8% of the United States population, has been recognized as a risk for MRSA SSTI (45). 

As indicated, only *aroA* type N was detected among the isolates originated from urinary tract infections. This type might be more adapted to the urinary tract and has special properties that play important role in its physiopathology. Baba-Moussa *et al* (2008) concluded that staphylococcal toxins and adhesion factors may be involved in the pathogenesis of *S. aureus* isolated from urinary tract infections (UTIs) (46). *In vitro*, S*. aureus* is able to attach to and aggregate onto uroepithelial cells through glycoproteins found in bladder mucin, like GP51, which is significantly increased in the presence of UTI (47). *S. aureus* harboring the Panton-Valentine Leukocidin (PVL) gene has been detected in urine, and this gene has been implicated in many different types of infections, including UTIs (48). Park *et al* (2008), believed that the *icaA* genes may enhance the adherence of *S. aureus* to host cells of the urinary tract, and may play a pathogenic role of UTI. Other speculation for this may be because of intermediate discriminatory power of *aroA*-gene based typing. In other study carried out by authors, this collection of *S. aureus* isolates were genotyped into six *coa* genotypes (C1-C6) using PCR-RFLP analysis. 

In that study, 16 urine isolates with the same *aroA* type (N) differentiated to four *coa* RFLP profile (unpublished data). However, the combined use of *coa* and *aroA* PCR-RFLP techniques demonstrated that most (71.4%) isolates of genotype N clustered separately within *coa* type C1 (unpublished data). This result rather supports the finding of El-Huneidi *et al* (2006), who confirmed that N is a separate genotype (23). Whole genome sequencing and comparative genomic analysis may be useful to characterize the molecular genetic features that distinguish N type optimized for skin and urinary tract infections in human from those that infect bovine hosts or are only infrequently recovered from human sources. As suggested by other researchers (22, 49), the correct epidemiological typing of *S. aureus* might require a combination of methods. 

## Conclusions

A very limited divergence between the isolates included in this study was described using *aroA*-gene based typing from two hospitals in the city of Urmia, Iran. However, the skin related isolates, were genetically divergence, may signifying cross-infection with *S. aureus* between humans and various sources such as environment. Here we also identified *aroaA* types which did not detected among *S. aureus* isolates recovered from UTIs. Most likely, the presence (combination) of virulence factors plays an important role in tissue specificity in *S. aureus* infections. This study also indicates that *aroA* genotypes vary not only from region to region, but also in individual host within a region. Further studies on these aspects from different regions of the country from time to time will help to monitor clonal dynamics of S*. aureus* in Iranian hospitals.
